# Emerging highly pathogenic H5 avian influenza viruses in France during winter 2015/16: phylogenetic analyses and markers for zoonotic potential

**DOI:** 10.2807/1560-7917.ES.2017.22.9.30473

**Published:** 2017-03-02

**Authors:** François-Xavier Briand, Audrey Schmitz, Katell Ogor, Aurélie Le Prioux, Cécile Guillou-Cloarec, Carole Guillemoto, Chantal Allée, Marie-Odile Le Bras, Edouard Hirchaud, Hélène Quenault, Fabrice Touzain, Martine Cherbonnel-Pansart, Evelyne Lemaitre, Céline Courtillon, Hélène Gares, Patrick Daniel, Alexandre Fediaevsky, Pascale Massin, Yannick Blanchard, Nicolas Eterradossi, Sylvie van der Werf, Véronique Jestin, Eric Niqueux

**Affiliations:** 1 Anses, Unité VIPAC - LNR influenza aviaire, Ploufragan, France; 2 Université Bretagne-Loire, Rennes, France; 3 Anses, Unité Génétique Virale et Biosécurité, Ploufragan, France; 4 Laboratoire Départemental d’Analyses et de Recherche, Coulounieix Chamiers, France; 5 Laboratoire des Pyrénées et des Landes, Mont-de-Marsan, France; 6 Direction Générale de l’Alimentation - MAAF, Paris, France; 7 Institut Pasteur, Unité Génétique Moléculaire des Virus à ARN - CNR grippe, Paris, France; 8 Anses – Groupe d’Experts Spécialisé Santé Animale et Bien-être Animal, Maisons-Alfort, France

**Keywords:** H5, Avian influenza, highly pathogenic, Zoonotic Potential, phylogeny

## Abstract

Several new highly pathogenic (HP) H5 avian influenza virus (AIV) have been detected in poultry farms from south-western France since November 2015, among which an HP H5N1. The zoonotic potential and origin of these AIVs immediately became matters of concern. One virus of each subtype H5N1 (150169a), H5N2 (150233) and H5N9 (150236) was characterised. All proved highly pathogenic for poultry as demonstrated molecularly by the presence of a polybasic cleavage site in their HA protein – with a sequence (HQRRKR/GLF) previously unknown among avian H5 HPAI viruses – or experimentally by the in vivo demonstration of an intravenous pathogenicity index of 2.9 for the H5N1 HP isolate. Phylogenetic analyses based on the full genomes obtained by NGS confirmed that the eight viral segments of the three isolates were all part of avian Eurasian phylogenetic lineage but differed from the Gs/Gd/1/96-like lineage. The study of the genetic characteristics at specific amino acid positions relevant for modulating the adaptation to and the virulence for mammals showed that presently, these viruses possess most molecular features characteristic of AIV and lack some major characteristics required for efficient respiratory transmission to or between humans. The three isolates are therefore predicted to have no significant pandemic potential.

## Introduction

On 24 November 2015, a highly pathogenic (HP) H5N1 avian influenza (AI) outbreak was confirmed by the French National Reference Laboratory (NRL) for avian influenza, in backyard layers and chickens in the Dordogne department, south-western France. From November 2015 to August 2016, a total of 80 highly pathogenic viruses have been identified, belonging to three different subtypes (H5N1, H5N2, H5N9).

In France, before 2015, the last H5 HPAI event was limited to wild swans *(Cygnus olor)* and mallards *(Anas platyrhynchos)*, in the eastern part of France during spring 2006 and summer 2007. It occurred almost concomitantly with outbreaks in wild birds and/or poultry in central Europe. The French HPH5 viruses isolated in 2006 and 2007 belonged to the A/goose/Guangdong/1/1996 (Gs/Gd/1/96-like) lineage (clade 2.2 subgroup), as did the 2007 viruses from central Europe) [1-5]. Until 2014, H5N1 HPAI viruses belonging to the Gs/Gd/1/96-like lineage have been maintained in south-east Asia, the Middle East and Egypt, in different locations and their haemagglutinin (HA) genes evolved continuously into a wide range of clades and subclades. They were reintroduced in West Africa and eastern Europe in 2015, and reassorted extensively, which in 2014 led to the emergence of new H5N6 and H5N8 HPAI subtypes (known as clade 2.3.4.4) in south-east Asia. The 2.3.4.4 H5N8 HPAI viruses spread to Europe (mainly the northern part) and to North America in late 2014. They are currently widespread in wildlife and poultry farms in the European Union (EU). In North America, they further reassorted as H5N2 and H5N1 HPAI, by incorporating neuraminidase (NA) genes of AIVs belonging to the American lineage. In the EU, recent reports also indicate reassortment as H5N5 HPAI [6]. However, no Gs/Gd/1/96-like viruses have been detected in France before November 2015, except for the 2006 and 2007 outbreaks mentioned above [7-12].

In contrast, low pathogenic (LP) H5N1, as well as H5N2 and H5N3 viruses, were detected in France in poultry, predominantly in domestic ducks, on several occasions [13-15], albeit seldom considering the fact that antibodies against H5 were regularly detected in sera of apparently healthy domestic ducks and geese during annual serological surveys [16-19].

The relationships between the H5 HPAI viruses detected in November 2015 and other H5 HPAI and LPAI viruses, including H5N1 viruses previously detected in France and worldwide, as well as their zoonotic potential and origin were immediately matters of concern. Based on whole genome sequences established by next generation sequencing (NGS), this paper focuses on the phylogenetic relatedness of these newly isolated viruses and on their genetic characteristics at specific amino acid positions already reported as relevant for cross-species transmission, adaptation to and virulence for mammals, including humans.

## Methods

Three AIV subtypes were detected in south-western France during the winter 2015/16 (H5N1, H5N2, H5N9). One representative of each subtype is described in the present paper.

### Outbreak description

The first virus was isolated from an affected chicken in a suspected outbreak of avian influenza virus infections (150169a; H5N1) declared to the local French animal health services on 19 November 2015. The affected flock was a backyard of 32 layer hens and broiler chickens, 9 to 10 months of age, located in Biras, Dordogne department. Die-off without any previous clinical signs caused a mortality rate of 69%. Post-mortem examination performed at the local veterinary diagnosis laboratory showed sub-cutaneous oedema of the head, neck and breast.

The second virus investigated here (150233; H5N2) was obtained from a duck on a farm located in Manciet, Gers departement, maintaining 8,300 ducks for fattening and fatty liver production, where 3% mortality was observed on 8 December 2015.

The third virus (150236; H5N9) was from a duck in Arrosès, Pyrénées-Atlantiques departement, where a flock of 500 ducks raised for fatty liver production experienced a 5% mortality on 9 December 2015.

### Detection, subtyping and molecular pathotyping

For each outbreak, RNA was extracted (RNeasy mini kit, Qiagen) from cloacal and oropharyngeal pools of five samples from dead birds. Samples were tested with the screening M-gene and H5 rRT-PCRs [20] by district laboratories. H5-positive samples were sent to NRL for further characterisation. The nucleotide (nt) sequences encompassing the cleavage site in the HA genes were amplified by the J3/B2a or Kha1/Kha3 RT-PCRs [21]. Similarly, a portion of the NA gene was amplified by Pan NA RT-PCR [22]. PCR products were sequenced using the PCR primers. Subtype determination was performed by BLAST against sequences of the Influenza virus resource database [23]. The theoretical pathotype of the viruses was inferred from the cleavage site sequences according to the World Organisation for Animal Health (OIE) / Food and Agriculture Organisation (FAO) Network of expertise on animal Influenza (OFFLU) [24].

### Virus isolation and in vivo characterisation

Virus isolation was performed from individual tracheal and/or cloacal swabs, on 9-day-old specific pathogen free (SPF) eggs, and was positive after the first passage. Viral pathogenicity was confirmed through determination of the HA cleavage site sequence and in vivo by inoculation of the H5N1HP 150169a isolate to 6.5-week-old SPF chickens, to establish the intravenous pathogenicity index (IVPI). All methods were conducted according to the OIE manual of standards for diagnostic tests and vaccines [25]. The IVPI experiment was performed according to international standards and was approved by the French Agency for Food, Environmental and Occupational Health and Safety (Anses)/National veterinary school of Alfort (ENVA)/Paris-est Créteil University (UPEC) Ethics Committee (no.14–060–18/11/14–6).

### Library preparation, whole genome sequencing and NGS data analysis

Total RNA (170–200ng) was extracted from infected allantoic fluid and was treated with DNase, then was depleted from rRNA. cDNA libraries were prepared using the Ion Total RNA-Seq Kit (Life Technologies, Carlsbad, California, United States (US)) according to a protocol adapted from supplier’s instructions (available upon request from the authors). The cDNA libraries were enriched then sequenced using the Ion Proton Sequencer and an Ion PI Chip v2 (Life Technologies). The resulting reads were cleaned with the Trimmomatic 0.32 software, then a Bowtie 2 alignment was performed on avian influenza genome references. The reads were down-sampled to fit a global coverage estimation of 80 x and were submitted to the SPAdes 3.1.1 de novo assembler. The de novo contigs were then submitted to BLAST on a local nt database. For each segment the best matches were selected for a Bowtie 2 alignment, which produces very clean and robust 5’ and 3’ ends, contrary to de novo assemblies of viral genomes for which 5’ and 3’ ends are sometimes incomplete. Finally, the de novo assemblies and the alignment on the references were compared and the strict identities of the de novo and aligned sequences were assessed.

For each virus, the consensus of the eight avian influenza segments was submitted to the GenBank database (accession numbers KU310444 to KU310451, KX014875 to KX014882 and KX014883 to KX014890 for isolates 150169, 150233 and 150336, respectively).

### Phylogenetic analysis

For each segment, the sequences were aligned with most of the closest full-length related sequences, as obtained by BLAST, and with genetic sequences selected as representative of the segment genetic diversity. Then, the neighbour-joining method based on the Kimura-2 parameter model was applied using the MEGA 6 software [26] to obtain phylogenetic trees with 1,000 bootstrap replicates.

### In silico analysis of molecular markers for transmission, replication and/or virulence in mammalian hosts

For the in silico prediction of the zoonotic potential of the new French viruses, the deduced amino acid sequences of viral proteins were analysed to search for the presence of residues previously known to be associated with increased transmission, replication, and/or virulence in mammalian hosts. The analysis was based on the inventory provided by the US Centers for Disease Control and Prevention (CDC) [27] and on a recent review by Neumann et al. [28].

## Results

### Virus isolation and in vivo pathotyping

Allantoic fluids with a haemagglutinating activity were collected from the inoculated embryonated eggs after the first passage of the three samples.

Isolates obtained from the allantoic fluids were further identified by HI test as H5 influenza viruses which were designated A/chicken/France/150169a/2015, A/duck/France/150233/2015 and A/duck/France/150236/2015 for the H5N1, H5N2 and H5N9 isolates, respectively. Because the HA genes of the three isolates were so similar, and in order to reduce the number of animal experiments, only the first virus isolate, the 150169a H5N1 virus, was tested in vivo for pathogenicity. Following intravenous inoculation, mortality was observed on the first day (3 of 10 birds died) and all inoculated birds had died on the second day. The IVPI was 2.9, close to its maximum value of 3, and higher than the regulatory threshold value of 1.2 required to declare the isolate highly pathogenic [25].

### Sequence analysis

Tables 1 and 2 present the closest genetically related avian influenza sequences, as identified by BLAST, and the percent nt identities between the three newly determined genomes, respectively. Although the three French HA sequences were close (nucleotide percent identities from 98.9 to 99.8), and shared a polybasic HQRRKR/GLF cleavage site not previously recognised in avian H5 HP viruses [24], their internal genes proved more distant with percent identities ranging from 93.1 to 99.5, and even more distant for the NA genes which belonged to the N1, N2 and N9 NA subtypes.

**Table 1:** GenBank accession number, full reference and percent nucleotide identity of the complete coding sequences of the eight genome segments of three avian influenza H5 viruses with their closest genetic relatives, France, November 2015

Segments	Closest relatives of H5N1 150169a virus	Closest relatives of H5N2 150233 virus	Closest relatives of H5N9 150236 virus
PB2	KM213385 A/ruddy turnstone/Iceland/2899/2013(H5N1) 98,2	KF918334 A/Italy/3/2013(H7N7) 97,5	KM213385 A/ruddy turnstone/Iceland/2899/2013(H5N1) 97,9
PB1	CY183997 A/mallard/Sweden/133546/2011(H10N4) 98,4	KP137828 A/harbour seal/Germany/1/2014(H10N7)) 97,5	CY041344 A/common eider/Netherlands/1/2006(H3N8) 97,2
PA	CY185470 A/common teal/Republic of Georgia/1/2011(H3N8) 98,2	CY184282 A/mallard/Sweden/100878/2009(H11N9) 98,3	KF874480 A/wild waterfowl/Dongting/C2383/2012(H1N2) 98,4
HA	KF462362 A/european teal/Novosibirsk/203/2011(H5N1) 96,4	KF462362 A/European teal/Novosibirsk/203/2011(H5N1)) 96,8	KF462362 A/European teal/Novosibirsk/203/2011(H5N1) 96,6
NP	CY185468 A/common teal/Republic of Georgia/1/2011(H3N8) 98,5	CY165689 A/mallard/Sweden/93211/2009(H4N6) 97,3	CY046143 A/mallard/France/061054/2006(H5N3) 96,7
NA	AIJ10960 A/ruddy turnstone/Iceland/2899/2013(H5N1) 98,2	CY185579 A/mallard/Republic of Georgia/13/2011(H6N2) 98,1	CY184263 A/mallard/Sweden/101011/2009(H11N9) 97,8
M	CY185341 A/mallard/Chany/425/2009(H4N6) 99	CY183800 A/mallard/Sweden/64476/2007(H10N4) 98,4	CY183800 A/mallard/Sweden/64476/2007(H10N4)) 98,5
NS	GQ907290 A/bar headed goose/Mongolia/143/2005(H12N3) 97,8	KF260032 A/common teal/Hong Kong/MPD322/2007(H11N9) 97,4	KF2600329 A/common teal/Hong Kong/MPD322/2007(H11N9) 97,3

HA: haemagglutinin; NA: neuraminidase.

**Table 2:** Percent nucleotide identity of the eight complete coding sequences between the three avian influenza H5 viruses, France, November 2015

	PB2	PB1	PA	HA	NP	M	NS
150169a (H5N1) to 150233 (H5N2)	96.6	94.5	93.1	98.9	95.5	95.6	98.6
150169a (H5N1) to 150236 (H5N9)	97.5	94.9	94.4	98.8	92.3	95.9	98.7
150233 (H5N2) to 150236 (H5N9)	96.4	93.3	93.4	99.8	91.6	99.5	99.2

HA: haemagglutinin; NA: neuraminidase.

**Figure 1:**
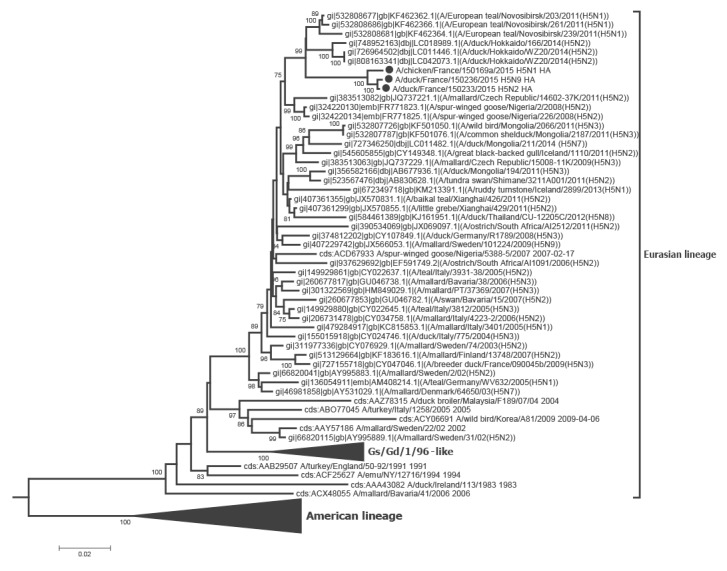
Phylogenetic tree of the H5 gene sequences, three avian influenza H5 viruses, France, November 2015

HA: haemagglutinin; HPAI: highly pathogenic avian influenza.

**Figure 2:**
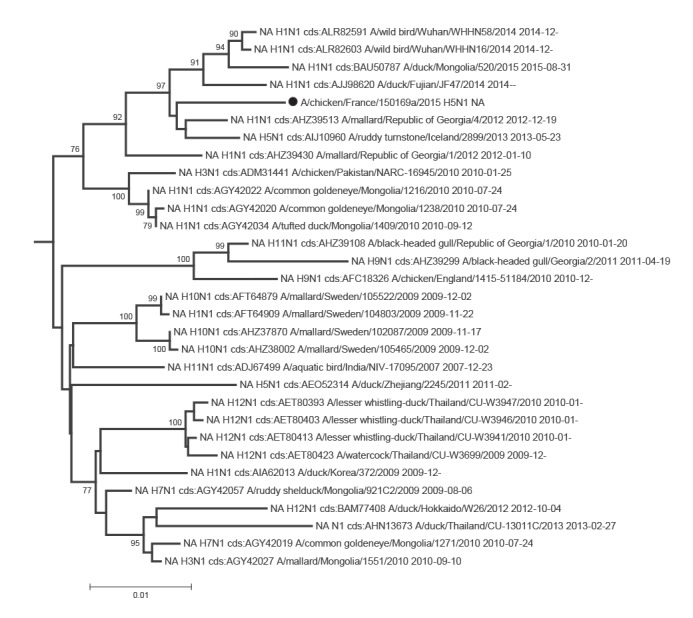
Phylogenetic trees of the neuraminidase gene sequences (N1 segment), three avian influenza H5 viruses, France, November 2015

HA: haemagglutinin; HP: highly pathogenic; M: matrix; NA: neuraminidase; nt: nucleotide.

**Figure 3:**
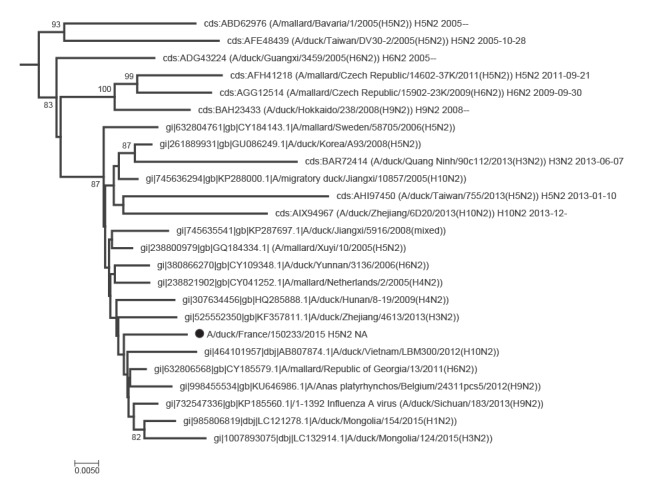
Phylogenetic trees of the neuraminidase gene sequences (N2 segment), three avian influenza H5 viruses, France, November 2015

HA: haemagglutinin; HP: highly pathogenic; M: matrix; NA: neuraminidase; nt: nucleotide.

**Figure 4:**
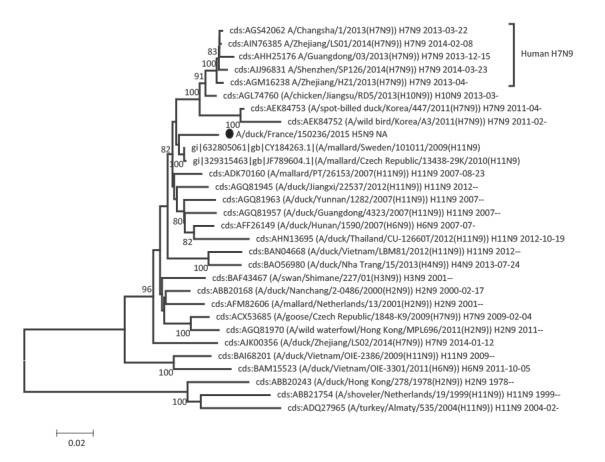
Phylogenetic trees of the neuraminidase gene sequences (N9 segment), three avian influenza H5 viruses, France, November 2015

HA: haemagglutinin; HP: highly pathogenic; M: matrix; NA: neuraminidase; nt: nucleotide.

Phylogenetic analyses of the eight segments of the French viruses confirmed that they were all part of the avian Eurasian lineage, as illustrated for the HA and NA genes in Figures 1, 2, 3, 4.

The complete coding sequences of the three H5 HPAI virus isolated from poultry in France were aligned with their closest related sequences as detected by BLAST, together with other sequences selected as representatives for more distant clusters. Then, the neighbour-joining method based on the Kimura-2 parameter model was applied using the MEGA 6 software [26] to obtain a phylogenetic tree with 1,000 bootstrap replicates. Only bootstrap values higher than 75% are shown. The HA sequences of French viruses are indicated with black dots. The tree was rooted by an H2 sequence.

The complete coding sequence of the three different neuraminidases of French H5 HP viruses were aligned with their closest related sequences as detected by BLAST, together with other sequences selected as representatives for more distant clusters. The neighbour-joining method based on the Kimura-2 parameter model was applied with the MEGA 6 software [26] to obtain a phylogenetic tree with 1,000 bootstrap replicates. Only bootstrap values higher than 75% are shown. The NA of French H5HP viruses are indicated by a black dot. The neuraminidase sequences from human viruses (H5N1 and H9N2) were not represented in the N1 and N2 trees respectively (nt distances between these sequences and the French sequences are too high).

The complete coding sequence of the three different neuraminidases of French H5 HP viruses were aligned with their closest related sequences as detected by BLAST, together with other sequences selected as representatives for more distant clusters. The neighbour-joining method based on the Kimura-2 parameter model was applied with the MEGA 6 software [26] to obtain a phylogenetic tree with 1,000 bootstrap replicates. Only bootstrap values higher than 75% are shown. The NA of French H5HP viruses are indicated by a black dot. The neuraminidase sequences from human viruses (H5N1 and H9N2) were not represented in the N1 and N2 trees respectively (nt distances between these sequences and the French sequences are too high).

The complete coding sequence of the three different neuraminidases of French H5 HP viruses were aligned with their closest related sequences as detected by BLAST, together with other sequences selected as representatives for more distant clusters. The neighbour-joining method based on the Kimura-2 parameter model was applied with the MEGA 6 software [26] to obtain a phylogenetic tree with 1,000 bootstrap replicates. Only bootstrap values higher than 75% are shown. The NA of French H5HP viruses are indicated by a black dot. The neuraminidase sequences from human viruses (H5N1 and H9N2) were not represented in the N1 and N2 trees respectively (nt distances between these sequences and the French sequences are too high).

The PB2 gene of the three French viruses belonged to a cluster including viruses from Europe, Gerogia, Iceland and notably one H9N2 virus previously detected in France (GenBank accession number: CY080412) and one avian HP H7N7 virus detected from Italian poultry workers with conjunctivitis [29].

For the PB1 gene, the sequence of the 150169a H5N1 virus was grouped mainly with viruses from Georgia, Mongolia and Sweden. The 150233 H5N2 virus sequence was grouped with a Belgian H5N2 and Chinese sequences and a sequence of an H10N7 virus isolated from a German harbor seal [30], whereas the sequence of the 150236 H5N9 virus was associated with sequences from various subtypes of AIVs from Sweden and the Netherlands.

The sequences of the PA segments clustered either with those of AIVs from Georgia, the Czech Republic, and the H10N7 virus from harbour seals [30] (150169a H5N1 isolate), or with virus sequences from Sweden and Norway (150233 H5N2 isolate), or with a group of sequences from Asian and Georgian viruses (150236 H5N9 isolate).

The HA phylogenetic analysis showed that the sequences of three French H5 HP clustered with those from Japanese, Mongolian and Russian viruses, but were not closely related to those of the Gs/Gd/1/96-like clades which all grouped in a separate cluster with a 100% bootstrap value (Figure 1).

As already observed with PB2, the NP sequences of 150169a (H5N1 virus) and 150233 (H5N2 virus) were grouped with those of viruses from Europe, Georgia and Iceland, again including the avian H7N7 isolated from poultry workers (A/Italy/3/2013) [29], whereas the NP gene of the French 150236 H5N9 HP was closely related with many other sequences from French H5 viruses isolated from 2006 to 2007 and with sequences from Dutch viruses.

The N1 gene sequence of 150169a H5N1 was related to sequences from viruses from Egypt, Georgia or Iceland. The Egyptian N1 sequence was from the avian H1N1 subtype, and not from H5N1 viruses with a zoonotic potential (Figure 2). The neuraminidase sequence from the 150233 H5N2 virus was close to an Asian N2 gene found in combination with several HA genes (H1, H3, H4, H5, H6, H9 and H10) and to a sequence from a Belgian H9N2 virus (Figure 3). The N9 sequence of isolate 150236 proved closest to several Swedish H11N9 sequences (Figure 4), and, although within a group with a significant bootstrap value, only distantly related with sequences from H7N9 viruses with a zoonotic potential.

For the M (matrix) gene sequences, only one large Eurasian cluster, including the three H5 HP viruses, exhibited a bootstrap value higher than 75%. This group included only four sequences of viruses from mammalian hosts, the aforementioned H7N7 and H10N7 viruses, as well as two H2N2 and H4N1 viruses detected in muskrat and swine, respectively [29-31].

Finally, the phylogenetic analysis of the three NS gene sequences indicated that they all belonged to a large cluster inside the A allele group, which contained sequences from African, Asian and European viruses, with only one sequence from a virus from a mammalian host, the swine H4N1 virus [31].

### Molecular markers for transmission, replication and/or virulence in mammalian hosts

The profiles of the three newly isolated viruses proved extremely similar in this respect, with only one position in the PB1-F2 protein differing between the three viruses.

Among the positions in the HA at which amino acid substitutions have been reported as potential determinants of host-range or of virulence for mammals, five were found in the HA of the new viruses (N94, N159, A160, P239, polybasic cleavage site). However, the HA of the isolated H5 HP viruses did not show the Q226L and G228S substitutions in the receptor-binding site of the HA that result in a switch of receptor binding preference from SAα2,3Gal to SAα2,6Gal [28].

Among the PB2, PB1 and PA polymerase complex proteins, PB2 has been shown to harbour major determinants of host-restriction and adaptation. None of these two major substitutions were found in any of the viruses investigated here. In PB1, substitutions shown to increase virulence in mice (V3A; N328K; N375S) or to contribute to airborne transmission in mammals (H99Y; I368V) were not present in the three new H5 HPAI viruses [32]. As shown in Table 3, at nine positions in PB2, three positions in PA and four positions in PB1 for which substitutions were shown to result in reduced polymerase activity or decreased virulence in mammals, the viruses exhibited residues typical of the vast majority (91.7%–100%) of European AIV. No amino acid changes associated with increased polymerase activity, virulence or transmission in mammals were present in the nucleoprotein. In the M protein, the viruses isolated in France since November 2015, as more than 99% of European AIV, harboured amino acids D30 and T215 associated with increased virulence for mice.

**Table 3:** Amino acid residues in different genes of highly pathogenic H5 viruses, at positions previously identified to promote transmission, replication (in vitro or in vivo) or pathogenicity in mammalian hosts, or associated with decreased sensitivity to antivirals, France, 2015^a^

Protein	Aa substitution^b^	Aa present in the French H5 viruses	The most represented residue for European AIV (% of this residue)^c^	Comments^d ^	PMID^e^
HA	D94N	N94	N94 (62.5)	Increased binding to alpha 2-6 receptor	19020946
	S159N; T160A	N159; A160	N159 (81.4); A160 (97.6)	Increased binding to alpha 2-6 receptor	20427525;19116267
	S239P	P239	P239 (99.4)	Slightly increased binding to alpha 2-6 receptor (aa 239 corresponds to aa 235 in H5 numbering)	21637809
	T160A	A160	A160 (97.6)	Increased airborne transmission in ferrets; increased binding to alpha2-6 receptor (aa 160 corresponds to aa 156 in H5 numbering)	22723413; 20427525
	Multibasic cleavage site	HQRRKR/GLF	Same sequence not found in other European viruses	Multiple basic residues in H5 viruses that are highly pathogenic for avian hosts (but unique sequence)	Same sequence not found in other European viruses
PB2	I63T	I63	I63 (99.2)	Decreased pathogenicity in mice in association with PB1 T677M	21367983
	L89V; G309D; T339K; R477G; I495V; A676T	V89; D309; K339; G477; V495; T676	V89 (99.7); D309 (99.5); K339 (91.7); G477 (100); V495 (99.4); T676 (98.3)	Increased polymerase activity in mouse cells	19393699
	R368Q; Q447H	R368; Q447	R368 (91.9); Q447 (99.9)	Reduced virulence (lethality in mice) and conferred histologic alteration in the lungs, liver and brain of ferrets	16533883; 15681421
PB1	K207R	K207	K207 (100)	Decreased polymerase activity	17553873
	Y436H	Y436	Y436 (99.9)	Decreased virulence in ducks, mice and ferrets	17553873
	V473L	V473	V473 (99.5)	Decreased polymerase activity in mammalian cells and mice	22090209
	T677M	T677	T677 (99.9)	Increased polymerase activity in vitro; reduced replication efficiency; decreased virulence in mice in association with PB2 I63T	21367983
PB1-F2	N66S	S66 (150169a); N66 (150233); PB1-F2 truncated (150236)	N66 (84,8); S66(15,1)	Increased replication efficiency in mice	21852950
PA	T515A	T515	T515 (99.8)	Decreased polymerase activity	17553873
	R266H; T515S	R266 T515	R266 (99.8) T515 (99.8)	Reduced polymerase activity in vitro	20211480
M1	N30D	D30	D30 (99.9)	Increased virulence in mice	19117585
	T215A	A215	A215 (99.9)	Increased virulence in mice	19117585
NS1	P42S	S42	S42 (66.0)	Increased virulence (lethality in mice and the systemic spread of infection); affected IFN pathway	18032512
	E92D	D92	D92 (99.8)	Cytokine resistance using antiviral activity assay	12195436
	L103F; I106M	F103; M106	F103 (65.7); M106 (99.8)	Increased virulence compared to WT in mice	19052083; 21593152
	N205S	S205	S205 (64.9)	Implicated in high virulence in ferrets	20862325
	227-230 (presence of PDZ ligand domain)	Amino acid motif (ESEV)	Amino acid motif (ESEV) (>80)	Amino acid motif (ESEV) increased virulence and pathogenicity in mice	18334632
NS2	T48A	A48	A48 (66.2)	Implicated in high virulence in ferrets	20862325

Aa: amino acids; AIV: avian influenza viruses; HA: haemagglutinin; IFN: Interferon; M: matrix; NA: neuraminidase; WT. : wild type

^a^ Markers for mammalian host adaptation or antiviral resistance were retrieved from [27] and the automated annotation of the studied sequence using the Influenza Research database [37].

^b^ H3 numbering for HA gene and numbering from the first methionine residue for other genes. Only positions where the sequence of French H5 HP viruses is consistent with a tropism for mammalian hosts are shown. For a more complete version with all positions shown, see ANSES table [12].

^c^ Percentage based on all complete protein sequences from viruses (all subtypes included except for the H5 and N1 genes) isolated in Europe and available in the Influenza Research database [37].

^d^ Comments on the biological effect of the studied set of mutations as per [27,37], except authors' comment for the HA cleavage site.

^e^ PMID numbers correspond to PubMed identifiers of cited references.

In the PB1-F2 of the 150169a H5N1 virus, the N66S substitution is observed as in HPAI H5N1 (Gs/Gd/1/96-like) and the 1918 pandemic virus. This substitution was not observed in the 150233 H5N2 or 150236 H5N9 viruses, the latter apparently exhibiting a truncated PB1-F2 ORF with a premature stop codon at position 26.

The NS1 protein is a major antagonist of the antiviral host responses. Among substitutions in NS1 associated with enhanced interferon antagonistic activity or that contribute to increased virulence in mammals, the P42S, E92D, L103F and I106M, and N205S mutations were present in the NS1 of these three French HP viruses as in the majority (> 65%) of European AIV. Furthermore, the ESEV PDZ-ligand domain, identified as an important virulence determinant, was present at the C-terminus of NS1 of the three French H5 viruses, as for more than 80% of European AIV.

Based on known substitutions in the M2 or the N1 that confer resistance or reduced susceptibility to antivirals, it could be considered that the French viruses are sensitive to both M2-blockers (amantadine, rimantadine) and neuraminidase inhibitors (zanamivir, oseltamivir, peramivir).

## Discussion

The unusual cleavage site corresponding to HP viruses observed in the November/December 2015 AIV circulation episode indicated that the acquisition of multiple basic residues did not occur by insertions as observed in the H5 HA from Gs/Gd/96-like viruses, but rather by substitution. Between positions 1009 to 1035 of the H5 encoding region, at least five nt substitutions were observed between the new French H5 HP and the closest H5 sequences. In addition, the full genome sequencing by NGS of the three French H5 HP virus isolates confirmed the presence of three different NA subtypes. The existence of reassortment events can be directly inferred from the finding that three different neuraminidases (N1, N2 and N9) were associated with very similar and original H5 HP sequences. This result seems to be confirmed by the phylogenetic analyses of the internal genes. Indeed, the internal genes of the three H5 HP viruses were not always directly closely related (higher percent identities in Table 1, as compared with Table 2). This demonstrates that the three viruses were not simply derived from a single H5HP ancestor through reassortment events leading to the acquisition of three different neuraminidases. More detailed analyses of the reassortment events will be made when other full genomes are sequenced, including non HP influenza viruses detected in the same area during the virological surveillance of the epizootic, which could have acted as partners in the reassortment events. Unfortunately, this research has been postponed due to the need to investigate the subsequent 2016/17 AIV circulation episode (due to H5N8 HPAI); in spite of intense virological surveillance, this investigation did not detect circulation of the 2015/16 H5 HPAI viruses.

Overall, based on the analysis of the sequences, only few residues that may increase transmission, replication and/or virulence in mammalian hosts were detected in the viruses analysed here. These residues were shared by the majority of other contemporary AIVs. To what extent the observed substitutions in the HA (N94, N159, A160, P234 and poly basic cleavage site) could contribute to the ability to bind the human SAα2,6Gal receptors in addition to avian SAα2,3Gal receptors needs to be evaluated in receptor-binding assays [33]. Such feature could potentially contribute to the ability of the virus to bind to both the upper and lower respiratory tract in humans. However, as for other European AIV including HPAI viruses, the polymerase complex proteins, the NP and the M1 of these viruses lack the major features associated with increased efficiency of replication in mammals. This does not preclude the possibility that under particular circumstances e.g. massive exposure or individual genetic susceptibility, infection in humans might occur and result in severe infections. Indeed, these viruses exhibit a multibasic cleavage site in their HA, that provides potential for systemic spread, and determinants in PB1-F2 and NS1 associated with an increased virulence in mammals and/or with the ability to antagonise the antiviral host response more efficiently.

However, the viruses did not exhibit the combinations of mutations found to be required for respiratory droplet transmission in ferrets [32,34,35]. These include (i) mutations in the HA that allow H5 HA binding to the SAα2,6Gal receptor as observed in human influenza viruses (N224K, Q226L, G228S); (ii) mutations resulting in the loss of a glycosylation site at position 158–160 in the HA that favours binding to the human SAα2,6Gal receptors; (iii) mutations at the HA trimer interface (H110Y; T318I) that increase the stability of the HA and result in a reduction of the optimal pH at which the conformational change required for fusion occurs; (iv) mutations in PB2 (E627K and D701N substitutions were considered as major determinants of adaptation to mammals [35,36]) and PB1 (H99Y) that ensure efficient viral replication in mammalian cells.

Hence, even in the very unlikely event of human infection with the 2015/16 H5 HPAIV, further human-to-human transmission is not anticipated and the pandemic potential of these viruses can be considered to be negligible.
